# Consumers’ Concerns Regarding Dining Out and Food Hygiene During the COVID-19 Pandemic in Japan: A Web-Based Questionnaire Survey

**DOI:** 10.7759/cureus.84923

**Published:** 2025-05-27

**Authors:** Shinya Matsumoto, Yoshiyuki Kanagawa, Kiwamu Nagoshi, Tomoaki Imamura, Manabu Akahane

**Affiliations:** 1 Department of Environmental Medicine and Public Health, Shimane University Faculty of Medicine, Izumo, JPN; 2 Department of Public Health, Health Management and Policy, Nara Medical University, Kashihara, JPN; 3 Department of Health and Welfare Services, National Institute of Public Health, Wako, JPN

**Keywords:** covid-19, dining industry, eating out, food safety, internet panel survey

## Abstract

Background

During a global infectious disease pandemic, governments impose varying levels of restrictions on daily life, including social activities, travel, and dining out. Understanding how these restrictions during the coronavirus disease 2019 (COVID-19) pandemic affected people's lifestyles and behaviors, especially regarding food and eating out, is essential for preparing effective countermeasures in future pandemics. The infection was believed to spread through droplets, with the first cluster reported on a pleasure boat. To prevent transmission, the government advised avoiding the "three Cs" (closed spaces, crowded places, and close-contact settings), increasing public anxiety about eating at restaurants.

Objective

This study aimed to examine the impact of the COVID-19 pandemic on consumer behavior and attitudes toward food and eating out in Japan, focusing on factors associated with dining-related anxiety.

Methods

An online survey was conducted in January 2021 with 1,442 Japanese participants aged 10-70 years. The survey collected data on dining habits, food safety concerns, and demographic information, including personality traits. Principal component analysis was applied to responses regarding "concerns about eating during the COVID-19 pandemic." Univariate and multivariate regression analyses were used to extract key factors contributing to these concerns.

Results

The study found that people were more anxious about eating at restaurants than at home or high-end establishments. Buffet-style meals, drink bars, and unwrapped disposable chopsticks were perceived as particularly risky. The first principal component of the principal component analysis had the same signs. The second principal component appeared at the opposite ends of the scale, with homes and buffets, event venues, and bars. Univariate linear regression analysis revealed items related to COVID-19. Multivariate linear regression showed not only items related to infection but also items related to food poisoning.

Conclusions

In Japan, the COVID-19 pandemic has significantly affected consumer behaviors and attitudes toward food and dining. While concerns about eating at home and high-end restaurants were relatively low, anxiety about buffet-style dining, drink bars, and unwrapped chopsticks was higher. Individuals concerned about eating out were more likely to prioritize infection control and food safety measures. Overall, the pandemic has led to a decline in restaurant visits and to preferences for home-cooked meals. Understanding these behavioral changes is crucial for the food industry to align with evolving consumer expectations in the post-pandemic era.

## Introduction

Since the end of 2019, coronavirus disease 2019 (COVID-19) has spread globally, causing numerous deaths and leading to restrictions on economic activity [1]. The virus reached Japan in early 2020, prompting initial restrictions on social activities due to uncertainties about its characteristics [2]. Travel abroad was restricted, and working from home was recommended, except for essential workers. The COVID-19 pandemic has profoundly impacted various aspects worldwide, significantly altering people’s behavior and perceptions [3,4]. Cancer screenings were scaled back, and older people went out less frequently. It is predicted that cancer mortality rates will increase in the future [5], and there are concerns about increased frailty in older adults [6].

The pandemic also intensified concerns about food safety and dining practices. As the pandemic spread, governments imposed restrictions that affected routines, including dining habits and hygiene practices [7]. Restrictions on movement, social distancing measures, and heightened awareness of viral transmission have led to a shift in consumer attitudes toward eating out and food safety [8]. Consumption patterns shifted with changes in the frequency and amount of snacks, alcohol, vegetables, and fruits consumed [9-12]. Food delivery services increased as restaurants and other food and beverage businesses faced restrictions. This shows that COVID-19 has had a significant impact not only on consumer behaviors related to eating out and food consumption but also on the entire dining industry and food supply chain [8], encompassing stages from food production to distribution.

COVID-19 primarily spreads through droplets. In droplet infection, the virus is transmitted when saliva or other bodily fluids containing the virus land on mucous membranes. Individuals can also become infected by touching their mucous membranes after contact with contaminated surfaces [13]. In Japan, the first cluster was reported on a houseboat (a boat with a covered room for sightseeing on a river or seaside that often serves food and drink). Other cluster cases were also reported in restaurants and karaoke [14]. Avoiding the "three Cs (closed spaces, crowded places, and close-contact settings)" has been suggested and recommended by the Japanese government. However, restaurants have been cited as an example of a place where the "three Cs" are likely to occur, and many people feel anxious about eating at restaurants. Consequently, the frequency of eating out decreased. Consumers' awareness and behavior regarding food and eating out may have changed significantly in Japan due to the COVID-19 pandemic.

This study aimed to explore changes in consumer behavior and attitudes toward food and eating out during the COVID-19 pandemic and to identify factors associated with anxiety about eating out. This study mainly focuses on hygiene and food safety during a pandemic, specifically examining consumer attitudes and behavioral intentions toward food during the COVID-19 pandemic. The aim is to identify findings that may be useful in the event of a future pandemic. These findings could have important implications for controlling the spread of infection and avoiding unnecessary or overly burdensome lifestyle restrictions during emergency situations. Based on the results of this study, it is hoped that a better understanding of consumer behavior and attitudes during a public health crisis will support timely and effective risk communication, not only to the food and beverage industry but also to the general public.

## Materials and methods

Study design and setting

The questionnaire survey was outsourced to an internet research company (Macromill, Inc.) and conducted online between January 27 and 28, 2021. A state of emergency was in effect when the survey was conducted. The survey targeted 1,442 males and females aged 10-70 years, with equal sex representation across all age groups.

Questionnaire items

The survey covered the following major categories with more detailed questions for each category: (1) What do you consider important when purchasing food? (2) Do you think the following are important for preventing food poisoning at home? (3) Do you think the following are "unsanitary" when eating at restaurants? (4) How worried are you about eating in the following places and formats during the COVID-19 pandemic?

Sex, age, residential area, and other demographic characteristics were collected as monitoring information data. In addition to personality, hygiene measures, information gathering, safety awareness, and brand awareness (electrical appliances, food, and clothing) were also collected.

For each item (with some exceptions), respondents were asked to respond using a six-point scale: “Completely disagree,” “Disagree,” “Somewhat disagree,” “Somewhat agree,” “Agree,” and “Strongly agree.” The questionnaire is shown in Appendix A.

Data analysis

First, we conducted a simple tabulation of responses to the following questions: "Are you worried about eating in the following places and ways during the COVID-19 pandemic?" and "When eating at a restaurant during the COVID-19 pandemic, are you worried that the following things may increase the risk of infection?"

Second, a principal component analysis was conducted on the question, "Are you worried about eating in the following places and ways during the COVID-19 pandemic?" Subsequently, a univariate regression analysis was conducted using the principal component score from the first principal component as the objective variable, with other questionnaire items not included in the principal component analysis serving as explanatory variables. The items used as explanatory variables were "Personality," "Hygiene measures," "Information gathering," "Safety awareness," "Brand awareness (electrical appliances, food, clothing)," "Things to consider when purchasing food, and what you think is important for preventing food poisoning at home," and "Feeling that eating at a restaurant is 'unsanitary.'" Next, a multivariate linear regression analysis (stepwise method) was conducted using the principal component score of the first principal component as the objective variable and the following questionnaire items not used in the principal component analysis as explanatory variables. If variable selection is performed using multivariate linear regression with a stepwise method, variables that are not very similar (i.e., have different meanings) tend to be selected. Multivariate linear regression was also performed to make the results easier to interpret. The results were evaluated using the t-value output from the linear regression analysis model. The t-value expresses the degree of discrepancy between the results and chance and indicates whether a variable should be included in the model. We used R version 4.1.1 (R Core Team, Vienna, Austria) for analysis.

Ethical approval

This study was approved by the Ethics Committee of the National Institute of Public Health, Japan (approval number: NIPH-IBRA12302), and the participants provided informed consent (completed online) for data collection and storage.

## Results

Of the 1,442 participants (103 males and females across age groups) who completed all survey questions, the distribution of residential areas was as follows: Hokkaido, 4.6%; Tohoku region, 5.0%; Chubu region, 16.5%; Kanto region, 40.2%; Kinki region, 19.1%; Chugoku region, 4.4%; Shikoku region, 2.1%; Kyushu region, 8.0%. These values were close to the approximate population distribution.

**Figure 1:**
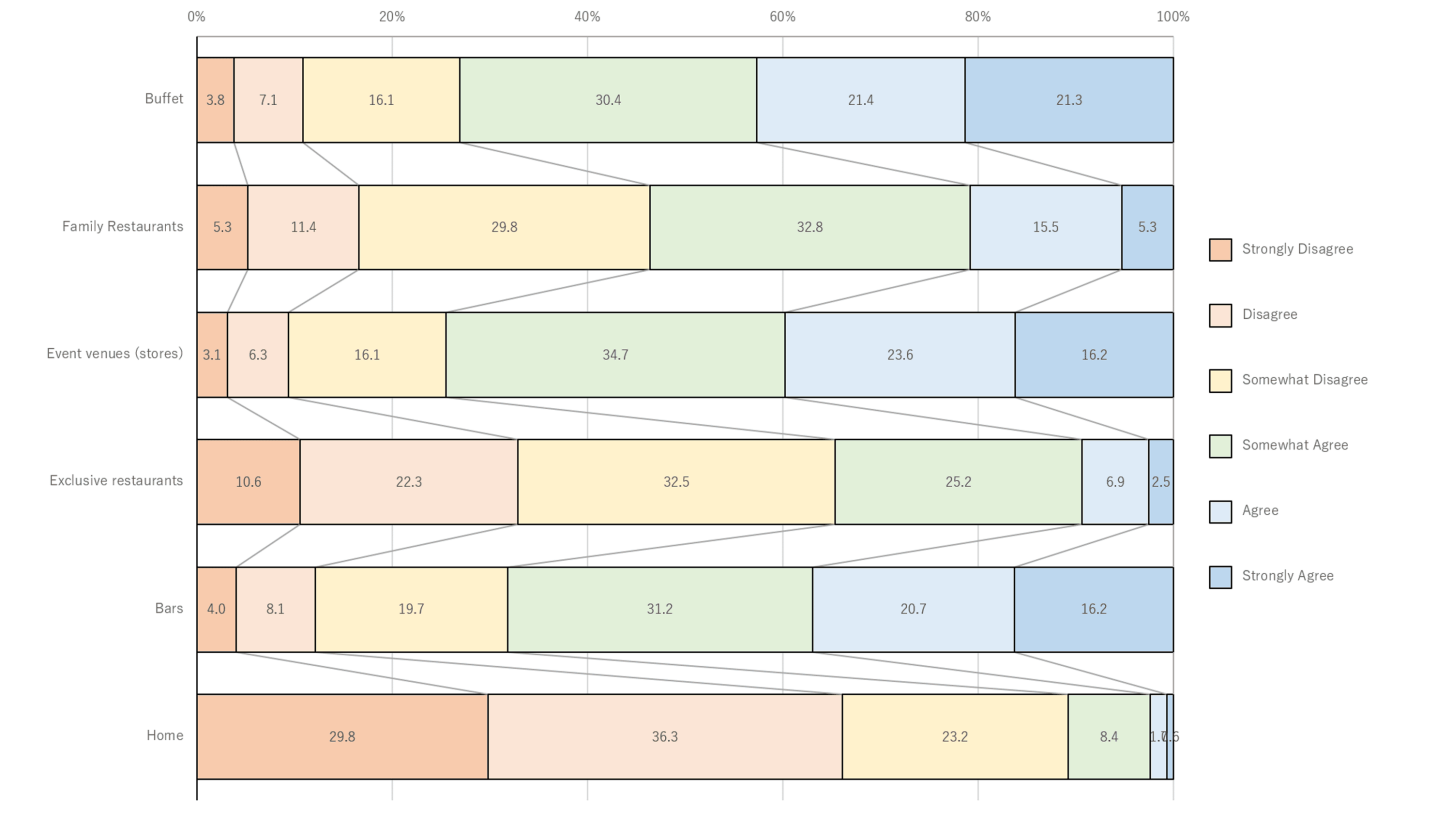
Percent of individuals concerned about eating at each location and business type during the COVID-19 pandemic Many people feel less anxious at home or in exclusive restaurants.

**Figure 2:**
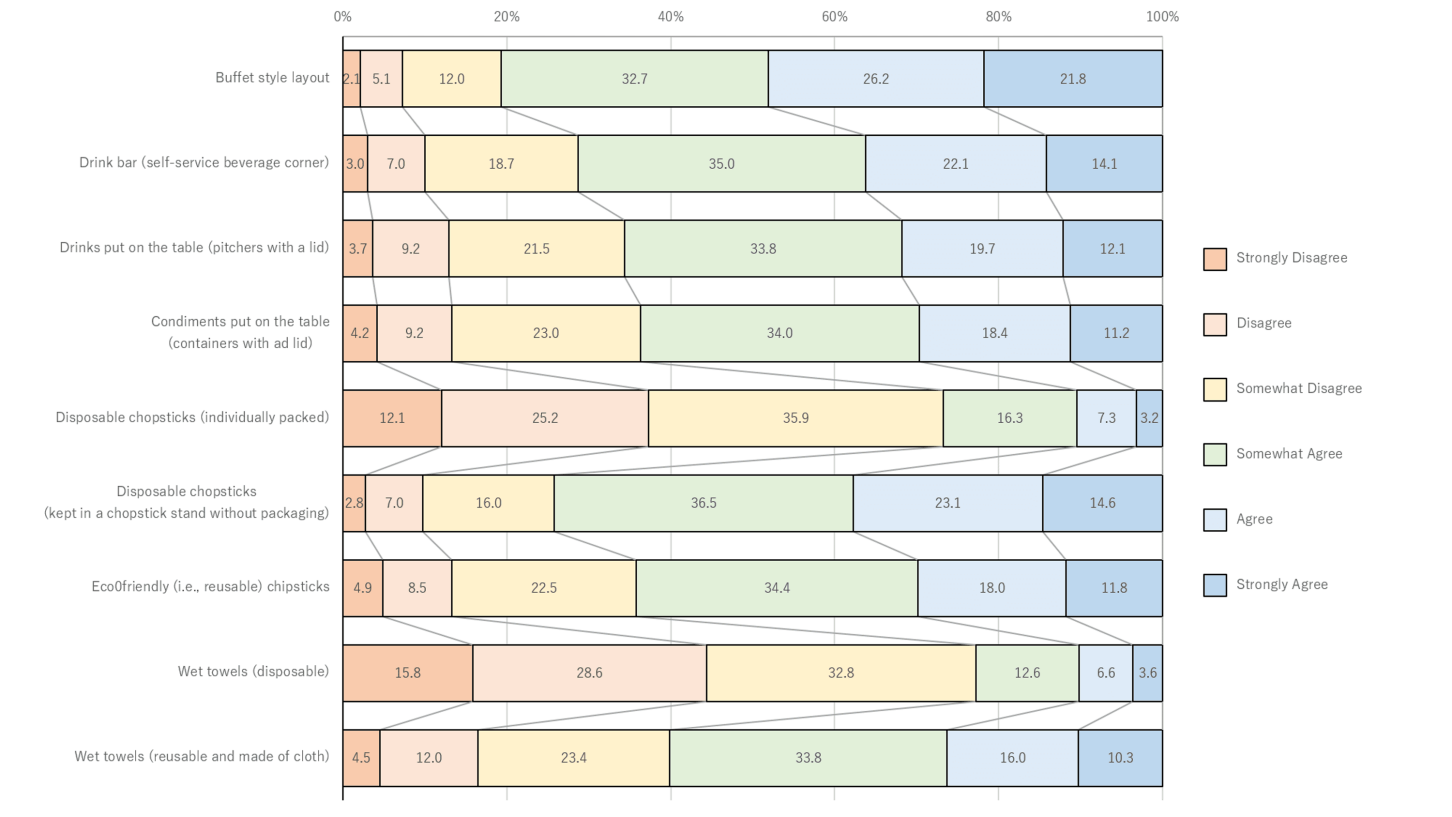
Percent of individuals concerned about increased infection risk from each of the following when eating in restaurants during a COVID-19 pandemic Many people don't feel particularly uneasy about individually packed chopsticks or disposable wet towels.

Figure 1 shows the results of the question, "To what extent do you feel anxious about eating in the following places and formats during the COVID-19 pandemic?" Concerns about eating at home or high-end restaurants tended to be lower than in other places. Figure 2 shows the results of the question, "When eating at a restaurant during the COVID-19 pandemic, were you worried that the following things would increase the risk of infection?" Individuals tended to feel anxious about buffet-style meals, drink bars, and disposable chopsticks (unwrapped: kept in a chopstick stand without packaging).

A principal component analysis was conducted on the six sub-questions in Q22: "How anxious do you feel about eating in the following places and formats during the COVID-19 pandemic?" In this study, we employed principal component analysis, a statistical technique that enables the extraction of common underlying components among variables. The most dominant component is identified as the first principal component, followed by the second most dominant as the second principal component.

Table 1 presents the factor loadings. Regarding the factor loadings resulting from the principal component analysis, the first principal component had the same sign, which is thought to indicate an overall sense of anxiety. The second principal component indicated a distinction between dining at home or in high-end restaurants and other restaurants (or locations/formats). This difference likely reflects the contrast in location and dining style, i.e., between eating at home/in a high-end dining restaurant and eating in restaurants other than those.

**Table 1:** Factor loading by principal component analysis The first principal component has the same sign and indicates an overall sense of anxiety. The second principal component has a mixture of positive and negative factors, indicating differences in anxiety depending on the dining location.

	Component 1	Component 2
Buffet	0.830	-0.285
Family restaurants	0.887	0.093
Event venues (stores)	0.836	-0.271
Exclusive restaurants	0.783	0.370
Bars	0.819	-0.222
Home	0.309	0.884

The results of the univariate linear regression analysis (top 10 variables) using the principal component score of the first principal component as the dependent variable are shown in Table 2. The top 10 variables were those that people felt were deemed unhygienic when eating at restaurants.

**Table 2:** Univariate linear regression results (top 10 variables) Questions related to Q16: "You consider the following as 'unhygienic' when eating at a restaurant": ranked highly.

No.	Question	t-value
1	You consider the following as “unhygienic” when eating at a restaurant: Buffet-style layout.	17.86
2	You consider the following as “unhygienic” when eating at a restaurant: Drinks placed on the table (pitchers with a lid).	16.64
3	You consider the following as “unhygienic” when eating at a restaurant: Drink bar (self-service beverage corner).	16.41
4	You consider the following as “unhygienic” when eating at a restaurant: Condiments placed on the table (containers with a lid).	15.77
5	You consider the following as “unhygienic” when eating at a restaurant: Disposable chopsticks (kept in a chopstick stand without packaging).	15.49
6	You consider the following as “unhygienic” when eating at a restaurant: Eco-friendly (i.e., reusable) chopsticks.	14.76
7	You consider the following as “unhygienic” when eating at a restaurant: Wet towels (reusable and made of cloth).	14.28
8	You consider the following when purchasing food. (Here, the rating would indicate the degree of consideration given.): Place (i.e., country) of production	10.095
9	The following are important to prevent food poisoning when eating at home. (“Expiry date” means the date by which food can be eaten safely, “best-before date” means the date by which food can be eaten without there being any change in its taste or quality, and “order of cooking” means the order in which you handle the ingredients when cooking chicken, seafood, and vegetables.): Order of cooking	9.819
10	You consider the following when purchasing food. (Here, the rating would indicate the degree of consideration given.): Foreign substance contamination countermeasures in the manufacturing process	9.514

The results of the multivariate linear regression analysis using the principal component score of the first principal component as the dependent variable are presented in Table 3. Related factors included the perception of unsanitary eating at restaurants, as well as what is important for preventing food poisoning at home.

**Table 3:** Multivariate linear regression (stepwise method) analysis results Perception of unhygienic food in restaurants and the importance of preventing foodborne illness at home were adopted as variables.

No.	Question	t-value
	Constant	-20.389
1	You consider the following as “unhygienic” when eating at a restaurant: Buffet-style layout.	7.466
2	When making a purchase, you consider product labels (marks, etc.) indicating that the product has been manufactured in a factory that adopts the following measures: Food defense measures.	2.261
3	You consider the following as “unhygienic” when eating at a restaurant: Wet towels (reusable and made of cloth).	2.593
4	The following are important to prevent food poisoning when eating at home: Order of cooking.	3.918
5	You consider the following as “unhygienic” when eating at a restaurant: Drinks placed on the table (pitchers with a lid).	2.693
6	You consider the following as “unhygienic” when eating at a restaurant: Eco-friendly (i.e., reusable) chopsticks.	1.943
7	When making a purchase, you consider product labels (marks, etc.) indicating that the product has been manufactured in a factory that adopts the following measures: Allergen labeling.	2.656
8	You consider the following as “unhygienic” when eating at a restaurant: Wet towels (disposable).	3.283
9	Do you try to obtain new information on the following topics?: Health	2.364
10	The following are important to prevent food poisoning when eating at home: Cleanliness of the place where you are cooking.	2.102
11	You consider the following as “unhygienic” when eating at a restaurant: Disposable chopsticks (kept in a chopstick stand without packaging).	2.016

## Discussion

This study revealed that, during the spread of COVID-19 infection, anxiety was lower for meals at home and high-end dining restaurants than in other venues. Participants expressed greater anxiety about buffet-style meals, drink bars, and unwrapped disposable chopsticks in Japan.

COVID-19 spreads primarily through droplets [13]. Specifically, droplets from direct contact with people through conversations or from surfaces are transferred through touch. These reports may have caused people to become highly anxious about the infectiousness and mode of COVID-19 transmission, leading them to focus on avoiding the "three C's" recommended by the government. Eating outside the home or in fine dining restaurants is a typical “three Cs”-related activity and may have contributed to people's anxiety. In the present study, the first principal component of the principal component analysis showed the same sign, indicating overall anxiety. In contrast, the second principal component revealed distinct trends for meals at home and high-end dining restaurants, suggesting that people’s anxiety was influenced by the intensity of their contact with others.

Filimonau et al. [15] conducted a study after the COVID-19 pandemic and reported how COVID-19 affected food consumption in British households. It found that during lockdown, home cooking increased, leading to more food waste. After the pandemic, people remained hesitant to eat out and preferred sustainable food at home, but not in restaurants. The findings suggest that foodservice providers should adopt hygiene measures and redesign their services to meet changing consumer expectations.

Univariate linear regression analysis showed that feeling unsanitary was one of the top factors. Multivariate linear regression analysis further highlighted concerns about hygiene in restaurants and the prevention of food poisoning at home as significant factors. At home, meals involve limited interaction with non-family members, while high-end dining restaurants reduce contact through widely spaced tables and private rooms. In contrast, casual dining venues typically have narrow gaps between tables, and quick-service restaurants may require customers to share tables with others.

Univariate linear regression analysis was used to evaluate the strength of connections between the dependent and explanatory variables. The first principal component, which places where people felt anxious during the COVID-19 pandemic, was the dependent variable. A comprehensive analysis of explanatory variables was performed; however, the top-ranking variable was strongly associated with services (Q16), prone to droplets sticking to objects, and increasing infection risk. Anxiety during the COVID-19 pandemic is believed to be closely related to infection risk. In a multivariate linear regression analysis (stepwise), variables with strong collinearity between explanatory variables were unlikely to be selected. Questionnaire items ranked high in the univariate linear regression but not selected in the multivariate linear regression were considered to represent concepts similar to the selected variables. Questionnaire items selected for the multivariate linear regression represented somewhat distant concepts. Items ranked high in the univariate linear regression and considered likely to transmit COVID-19 were selected first. Items related to food defense were also selected second. Food defense refers to the protection of food products from intentional contamination or adulteration, which can result from malicious acts [16,17] and is not directly related to COVID-19 infections. Cooking order was selected fourth. This was not related to eating out or to COVID-19 infection. It is a method to avoid regular food poisoning. People who are anxious about eating out are not only interested in infection but also in food defense and food hygiene.

The COVID-19 pandemic has profoundly impacted various aspects of daily life worldwide, significantly altering consumer behavior and perceptions, particularly concerning food safety and dining practices. As the pandemic spread, national governments imposed restrictions that affected people's routines, including dining habits and hygiene practices [7]. Restrictions on movement, social distancing measures, and heightened awareness of viral transmission have led to a shift in consumer attitudes toward eating out and food safety [8,12]. Before the pandemic, dining out was a common social activity and a frequent choice for many individuals. However, as COVID-19 began to spread, infection risk from going out increased, and this heightened anxiety influenced consumer behavior, making many people more cautious and changing their eating habits. The increased perception of the risks associated with eating out led to a greater preference for home-cooked meals and a significant decline in restaurant visits [18].

Consumers’ heightened focus on hygiene and safety has intensified during the pandemic, prompting a reevaluation of food-handling practices and the sanitation standards maintained by food establishments [19]. This increased vigilance is reflected in the consumer demand for enhanced safety measures, including stricter hygiene protocols and transparency in food preparation and handling processes [20]. The emphasis on hygiene has led to the implementation of new standards and practices within the food industry aimed at addressing consumer concerns and ensuring dining safety. In addition, the concept of food defense has gained renewed importance during the pandemic. Heightened awareness and concerns regarding food safety during the pandemic underscore the need for comprehensive food defense strategies. This includes securing supply chains, monitoring potential threats, and ensuring food product integrity throughout production and distribution processes. Integrating food defense with existing safety protocols is essential for maintaining consumer trust and safeguarding public health.

Limitations

This study has several limitations that must be acknowledged. First, this survey was conducted online, and the sample was selected based on age group and sex distribution; therefore, it did not consider the age distribution of the population in the country or place of residence. Second, the data were collected only from respondents registered with an internet research company, which may have introduced sample bias. Nonetheless, panel-based surveys have been widely used in recent years [21,22]. Third, the time frame of this study is limited. This study is based on data collected at a single point in time during the COVID-19 pandemic. Given the prolonged duration of the pandemic, it may be possible that social conditions and consumer attitudes varied throughout that period. At this point, further research is needed to examine how consumer attitudes have evolved as social conditions have recovered in the post-pandemic period.

## Conclusions

During the spread of COVID-19 in Japan, concerns about meals at home and in high-end restaurants were lower than in other dining settings. Greater anxiety was reported for buffet-style meals, drink bars, and unwrapped disposable chopsticks. Although dining at bars and restaurants is likely to resume once the pandemic subsides, lingering memories of anxiety and related concerns may prevent a complete return to pre-pandemic behaviors. The restaurant industry must adapt to these changes to meet evolving consumer expectations.

## Appendices

Appendix A 

**Table 4:** Questionnaire items The table shows the questionnaire items administered in this study. Q22 was used as the final dependent variable with principal component analysis. This questionnaire was prepared by M.A. and Y.K. *1: Strongly disagree/Disagree/Somewhat disagree/Somewhat agree/Agree/Strongly agree *2: Yes/No

ID	Question Statement	Choices
Q1	Please tell us about yourself.	
Q1S1	I am diligent.	*1
Q1S2	I am sociable.	*1
Q1S3	I have a strong sense of responsibility.	*1
Q1S4	I have a strong sense of morality.	*1
Q1S5	I am cooperative.	*1
Q1S6	I am honest.	*1
Q2	You practice the following often to maintain hygiene and cleanliness?	
Q2S1	Handwashing	*1
Q2S2	Gargling	*1
Q2S3	Wearing a mask	*1
Q2S4	Cleaning	*1
Q2S5	Organizing	*1
Q3	You try to obtain new information on the following topics?	
Q3S1	Food	*1
Q3S2	Health	*1
Q3S3	Environment	*1
Q3S4	Domestic situation	*1
Q3S5	International situation	*1
Q4	Please tell us about your thoughts on safety.	
Q4S1	I will pay additional fees to ensure safety.	*1
Q4S2	Terror acts do not occur in Japan.	*1
Q4S3	Inspection of belongings for entry into sports facilities is appropriate.	*1
Q4S4	Surveillance cameras ensure safety.	*1
Q4S5	Restrictions are necessary for safety.	*1
Q5	The following are important for you when buying electrical appliances. (Here, the rating would indicate the degree of importance.)	
Q5S1	Price	*1
Q5S2	Brand	*1
Q5S3	Manufactured domestically	*1
Q5S4	Reputation (word of mouth)	*1
Q5S5	Customer service/troubleshooting	*1
Q5S6	Safety	*1
Q6	6) The following are important for you when buying food. (Here, the rating would indicate the degree of importance.)	
Q6S1	Price	*1
Q6S2	Brand	*1
Q6S3	Manufactured domestically	*1
Q6S4	Reputation (word of mouth)	*1
Q6S5	Customer service/troubleshooting	*1
Q6S6	Safety	*1
Q7	The following are important for you when buying clothes. (Here, the rating would indicate the degree of importance.)	
Q7S1	Price	*1
Q7S2	Brand	*1
Q7S3	Manufactured domestically	*1
Q7S4	Reputation (word of mouth)	*1
Q7S5	Customer service/troubleshooting	*1
Q7S6	Safety	*1
Q8	Have you heard of the following terms?	
Q8S1	Food security and safety	*2
Q8S2	Food hygiene	*2
Q8S3	Intentional contamination of food	*2
Q8S4	Food terrorism	*2
Q8S5	Food defense	*2
Q9	The frozen food you purchased is contaminated with foreign substances (metal, hair, etc.). You…	
Q9S1	Eat it without worrying	*1
Q9S2	Contact the manufacturer	*1
Q9S3	Contact the shop from where you purchased it	*1
Q9S4	Upload it on SNS	*1
Q9S5	Dispose of it	*1
Q10	The frozen food you purchased has a bad (rotten, chemical, etc.) smell. You…	
Q10S1	Eat it without worrying	*1
Q10S2	Contact the shop from where you had ordered it	*1
Q10S3	Upload it on SNS	*1
Q10S4	Dispose of only the extra item(s)	*1
Q10S5	Dispose of all the items	*1
Q12	The following are important to prevent food poisoning when eating at home. (“Expiry date” means the date by which food can be eaten safely, “best-before date” means the date by which food can be eaten without there being any change in its taste or quality, and “order of cooking” means the order in which you handle the ingredients when cooking chicken, seafood, and vegetables.)	
Q12S1	Expiry date	*1
Q12S2	Best-before date	*1
Q12S3	Cold storage of ingredients	*1
Q12S4	Heat cooking	*1
Q12S5	Order of cooking	*1
Q12S6	Cleanliness of the place where you are cooking	*1
Q13	Considering that there have been instances of intentional contamination of food with foreign substances (metal, pesticide, etc.), the following types of food are at high risk. (“Intentional contamination of food with foreign substances” refers to introducing foreign substances (metal, pesticide, etc.) into food with malicious intent.)	
Q13S1	Food produced domestically by large domestic enterprises	*1
Q13S2	Food produced domestically by small and medium domestic enterprises	*1
Q13S3	Food produced overseas by large foreign enterprises	*1
Q13S4	Food produced overseas by small and medium foreign enterprises	*1
Q13S5	Food produced by privately managed domestic stores	*1
Q14	The food sold at the following places is at high risk of intentional contamination with foreign substances.	
Q14S1	Privately managed restaurants	*1
Q14S2	Chain restaurant outlets	*1
Q14S3	Exclusive restaurants	*1
Q14S4	Sports stadiums (baseball grounds, etc.)	*1
Q14S5	Kitchen cars/street stalls	*1
Q15	The drinks sold at the following places are at high risk of intentional contamination with foreign substances.	
Q15S1	Kitchen cars/street stalls	*1
Q15S2	Sports stadiums (baseball grounds, etc.)	*1
Q15S3	Vending machines	*1
Q15S4	Sold directly by vendors	*1
Q15S5	Chain restaurant outlets	*1
Q16	You consider the following as “unhygienic” when eating at a restaurant.	
Q16S1	Buffet style layout	*1
Q16S2	Drinks bar (self-service beverage corner)	*1
Q16S3	Drinks put on the table (pitchers with a lid)	*1
Q16S4	Condiments put on the table (containers with a lid)	*1
Q16S5	Disposable chopsticks (individually packed)	*1
Q16S6	Disposable chopsticks (kept in a chopstick stand without packaging)	*1
Q16S7	Eco-friendly (i.e., reusable) chopsticks	*1
Q16S8	Wet towels (disposable)	*1
Q16S9	Wet towels (reusable and made of cloth)	*1
Q17	You worry about the following at international sports events held in the summer in Japan. (“Intentional contamination of food with foreign substances” refers to introducing foreign substances (metal, pesticide, etc.) into food with malicious intent.)	
Q17S1	Heat stroke	*1
Q17S2	Mass food poisoning	*1
Q17S3	Intentional contamination of food with foreign substances	*1
Q17S4	Terror attack(s)	*1
Q17S5	The spread of COVID-19	*1
Q18	You consider the following when purchasing food. (Here, the rating would indicate the degree of consideration given.)	
Q18S1	Ingredient labeling	*1
Q18S2	Place (i.e., country) of production	*1
Q18S3	Hygiene control measures in the manufacturing process	*1
Q18S4	Foreign substance contamination countermeasures in the manufacturing process	*1
Q18S5	Expiry date/best-before date	*1
Q19	There have been several instances of intentional contamination of food with foreign substances/drugs in the past, and implementing measures to prevent it is called “food defense.” Considering this, such measures should be adopted for food manufactured/provided at the following places.	
Q19S1	Food factories	*1
Q19S2	Food service chain stores (restaurants, etc.)	*1
Q19S3	Retail stores (supermarkets, convenience stores, etc.)	*1
Q19S4	International sports event venues (Olympics, etc.)	*1
Q19S5	International political event venues (summits, etc.)	*1
Q20	Considering that there have been several incidents of intentional contamination of food with foreign substances (pesticide, etc.) in Japan, such incidents will recur in the future at the following places.	
Q20S1	Food factories	*1
Q20S2	Food service chain stores (restaurants, etc.)	*1
Q20S3	Retail stores (supermarkets, convenience stores, etc.)	*1
Q20S4	Food-related logistics facilities	*1
Q20S5	Event venues (sports, etc.)	*1
Q20S6	Stores selling imported food items	*1
Q20S7	Other (please specify)	*1
Q21	When making a purchase, you take into consideration product labels (marks, etc.) indicating that the product has been manufactured in a factory that adopts the following measures. (Food hygiene measures are measures to prevent food poisoning, while food defense measures are measures to prevent intentional contamination of food with foreign substances/drugs.)	
Q21S1	Food hygiene measures	*1
Q21S2	Food defense measures	*1
Q21S3	Allergen labeling	*1
Q21S4	Labeling of foods for specified health use (FOSHU)	*1
Q21S5	Vegetarian labeling	*1
Q22	You are anxious about eating in the following style/places amid the COVID-19 pandemic. (Here, the rating would indicate the degree of anxiousness.)	
Q22S1	Buffet	*1
Q22S2	Family restaurants	*1
Q22S3	Event venues (stores)	*1
Q22S4	Exclusive restaurants	*1
Q22S5	Bars	*1
Q22S6	Home	*1
Q23	You are worried the following will increase the risk of infection when eating at a restaurant amid the COVID-19 pandemic.	
Q23S1	Buffet style layout	*1
Q23S2	Drinks bar (self-service beverage corner)	*1
Q23S3	Drinks put on the table (pitchers with a lid)	*1
Q23S4	Condiments put on the table (containers with a lid)	*1
Q23S5	Disposable chopsticks (individually packed)	*1
Q23S6	Disposable chopsticks (kept in a chopstick stand without packaging)	*1
Q23S7	Eco-friendly (i.e., reusable) chopsticks	*1
Q23S8	Wet towels (disposable)	*1
Q23S9	Wet towels (reusable and made of cloth)	*1
